# A commentary on ‘Transversus thoracic muscle plane block for pain during cardiac surgery: a systematic review and meta-analysis’

**DOI:** 10.1097/JS9.0000000000001170

**Published:** 2024-02-12

**Authors:** Wei Zhong, Bo Zhao, Lei Liu, Gaoyuan Xi, Junhui Zhou

**Affiliations:** Department of Anesthesia, Henan Provincial Chest Hospital (Chest Hospital of Zhengzhou University), Zhengzhou, Henan, People’s Republic of China

*Dear Editor*,

It was with great interest that we read the article published in the *International Journal of Surgery*. Xue *et al*.^1^ performed a systematic review and meta-analysis of adult patients who got transversus thoracic muscle plane blocks (TTMPBs) or no block/sham block during heart surgery. After searching PubMed, Embase, Web of Science, CENTRAL, WanFang Data, and the China National Knowledge Infrastructure until June 2022, nine English-language studies involving 454 patients were chosen as appropriate for review. The study discovered that TTMPB during cardiac surgery likely lowers postoperative pain at rest and during movement, narcotic usage, ICU duration of stay, and nausea and vomiting.

The TTMPB is a relatively novel truncal interfascial plane block designed to anesthetize the anterior cutaneous branches of the thoracic intercostal nerves^2,3^. In TTMPB, a local anesthetic (LA) is delivered into the fascial plane, which is situated between the transversus thoracic and internal intercostal muscles, deeper. Therefore, by blocking the anterior chest wall, TTMPB has been shown in some studies to minimize initial poststernotomy discomfort and opiate usage. Previous studies have shown that by lowering postoperative insulin resistance, systemic inflammation, and sufentanil consumption, bilateral TTMPBs can improve postoperative recovery and offer good perioperative analgesia in patients undergoing open cardiac surgery. The findings of additional research point to the importance of TTMPB in reducing cognitive impairment following heart surgery^4^. Furthermore, because the needle tip’s path passes near the internal thoracic artery, pleura, and heart, the TTMPB may carry some risk for consequences (such as hemorrhage, pneumothorax, and systemic LA toxicity). However, no block-related side effects, including hemothorax, pneumothorax, and toxicity from LA, were noted in these cardiac surgery investigations.

Several factors should be considered: First, the report does not consider publication bias. However, the authors claimed to have searched PubMed, Embase, Web of Science, CENTRAL, WanFang Data, and the China National Knowledge Infrastructure for pertinent literature published before June 2022. Second, there was a great deal of heterogeneity in the analysis. The daily developed blocks, the type of surgery, the use of LA, and the non-homogeneous distribution of American Society of Anesthesiologists (ASA) II–III patients could all be contributing factors. Third, acute poststernotomy discomfort can last up to a week, even though the postoperative pain scores were only monitored for a full day.

To sum up, TTMPB during cardiac surgery likely lowers opioid usage, the length of stay in the ICU, the frequency of nausea and vomiting, and postoperative pain at rest and with movement. In the context of cardiac surgery, ultrasound-guided regional anesthesia is unquestionably a crucial analgesic approach since it preserves opioids, minimizing their neurological and hemodynamic influence while remaining unaffected by the patient’s coagulative status, allowing its use in non-elective procedures. Nevertheless, more excellent randomized controlled trials are needed to ascertain the effectiveness and safety of TTMPB in cardiac surgery. In cardiac surgery, an MCID (minimum clinically important difference) should be calculated in order to measure the impact of individual blocks in comparison to the gold standard of care^5^.

## Ethical approval

This manuscript is a comment. Don’t need ethical approval.

## Consent

This manuscript is a comment. Don’t need patient consent.

## Sources of funding

This work was supported by the 2020 Henan Province Medical Science and Technology Research Plan joint construction project (LHGJ20200216) and the National Key Clinical Specialty Construction Project of China.

## Author contribution

W.Z.: study concept or design, data collection, data analysis or interpretation, and writing the paper; B.Z., L.L., and G.X.: data collection; J.Z.: study concept or design, writing, and revising the paper.

## Conflicts of interest disclosure

There are no conflicts of interest in our commentary.

## Research registration unique identifying number (UIN)

This manuscript is a comment. Don’t need UIN.

## Guarantor

Junhui Zhou.

## Data availability statement

This manuscript is a comment. Don’t need a Data availability statement. However, all the data from the current study are publicly available.

## Provenance and peer review

This manuscript is a comment without being invited.
